# Oenological Characteristics of Four Non-*Saccharomyces* Yeast Strains With *β*-Glycosidase Activity

**DOI:** 10.3389/fmicb.2021.626920

**Published:** 2021-09-03

**Authors:** Tao Qin, Jing Liao, Yingyuan Zheng, Wenxia Zhang, Xiuyan Zhang

**Affiliations:** College of Food Science and Technology, Huazhong Agricultural University, Wuhan, China

**Keywords:** oenological characteristics, wine, flavor, *β*-glucosidase, non-*Saccharomyces* yeast

## Abstract

Non-*Saccharomyces* yeast with *β*-glucosidase activity might positively contribute to the flavor and quality of wines. The contribution of four non-*Saccharomyces* yeast strains *Issatchenkia terricola* SLY-4, *Pichia kudriavzevii* F2-24, *P. kudriavzevii* F2-16, and *Metschnikowia pulcherrima* HX-13 with *β*-glucosidase activity to the flavor and quality of wine making was studied. Compared with those of *S. cerevisiae* single fermentation, the four non-*Saccharomyces* yeast strains could grow and consume sugar completely with longer fermentation periods, and with no significantly negative effect on chemical characteristics of wines. Moreover, they produced lower content of C_6_ compounds, benzene derivative, and fatty acid ethyl ester compounds and higher content of terpene, *β*-ionone, higher alcohol, and acetate compounds. Different yeast strains produced different aroma compounds profiles. In general, the sensory evaluation score of adding non-*Saccharomyces* yeast-fermented wine was better than that of *S. cerevisiae*, and *I. terricola* SLY-4 fermentation received the highest one, followed by *P. kudriavzevii* F2-24, *P. kudriavzevii* F2-16, and *M. pulcherrima* HX-13 from high to low. The research results provide a theoretical basis for the breeding of non-*Saccharomyces* yeast and its application in wine making.

## Introduction

It is an established enological practice to use commercial *Saccharomyces cerevisiae* to ferment wine. Pure *S. cerevisiae* fermentation has an easy control fermentation process and a high consistency of product quality between batches, but it is easy to lead poor flavor complexity and varietal aroma characteristics of wine which are mainly contributed by varietal, fermentative, and aging aroma compounds (Pires et al., 2014).

At present, non-*Saccharomyces* yeast is widely accepted because of its ability to produce aroma compounds and other excellent brewing characteristics, which has been used in pure or mixed fermentation with *S. cerevisiae* to overcome the defect of imperfect wine flavor (Parker et al., 2017; Canonico et al., 2019; Plessis et al., 2019; Binati et al., 2020). The varietal aroma characteristics of wine are mainly contributed by the volatile varietal aroma compounds; however, these compounds often exist as non-volatile glycoside precursors and are odorless. The non-volatile glycosides can be hydrolyzed by *β*-glycosidases and released as volatile compounds with flavor (Cabrita et al., 2010; García-Carpintero et al., 2011). *β*-Glycosidase from different resources will affect the category and concentration of volatile varietal aroma compounds (Baffi et al., 2011). Generally, single fermentation of *S. cerevisiae* is weak in liberating these aroma precursors (Boscaino et al., 2019).

Several non-*Saccharomyces* yeast strains have been confirmed to have *β*-glucosidase activity, including *Hanseniaspora uvarum*, *Pichia fermentans*, *Pichia membranifaciens*, *Wickerhamomyces anomalus*, and *Rhodotorula mucilaginosa*, and they can improve the content of some volatile aroma compounds such as terpenes and benzene derivatives, imparting fruity and floral flavor profile to wine (Sabel et al., 2014; López et al., 2015, 2016; Ovalle et al., 2016; Ma et al., 2017; Sun et al., 2017). Four non-*Saccharomyces* yeast strains *I. terricola* SLY-4, *P. kudriavzevii* F2-24, *P. kudriavzevii* F2-16, and *M. pulcherrima* HX-13 were isolated from vineyards of the Helan Mountain region in Ningxia of China by our research group, which produced 98.51, 76.93, 62.72, and 47.95 U/l *β*-glucosidase activities, respectively (Wang et al., 2018). Adding crude extraction of *β*-glucosidases from *I. terricola* SLY-4, *P. kudriavzevii* F2-24, and *M. pulcherrima* HX-13 into must could increase the content of terpenes, esters, and fatty acids and enhance the fruity and floral aroma of wine fermented by *S. cerevisiae*. Wines using different crude *β*-glucosidase extractions presented distinct volatile compound profiles and varied typical flavor characteristics (Zhang et al., 2020). These results indicated that *β*-glucosidases from the four non-*Saccharomyces* yeast strains could enhance the content of aroma compounds with different profiles and improve the fruity and floral aroma of wine. However, the positive or negative effects of these four non-*Saccharomyces* yeast strains as main brewing yeast on wine-making are not yet known.

To investigate the effects of pure fermentations of *I. terricola* SLY-4, *P. kudriavzevii* F2-24, *P. kudriavzevii* F2-16, or *M. pulcherrima* HX-13 on flavor complexity and varietal aroma characteristics of wines, the process and the quality of their pure fermentations will be analyzed. Research results will provide some references of using non-*Saccharomyces* yeast strains to improve the flavor complexity and varietal characteristics of wines.

## Materials and Methods

### Strains and Medium

*I. terricola* SLY-4, *P. kudriavzevii* F2-24, *P. kudriavzevii* F2-16, and *M. pulcherrima* HX-13 were isolated from vineyards of the Helan Mountain region in Ningxia of China. They have been identified through sequence analysis of the 26S rDNA D1/D2 domain and kept in our lab. Reference strains were purchased from the China General Microbiological Culture Collection Center (CGMCC 2.3216 *Issatchenkia terricola*; CGMCC 2.454 *Pichia kudriavzevii*; CGMCC 2.3776 *Metschnikowia pulcherrima*). *Saccharomyces cerevisiae* was a commercial strain Actiflore^®^ F33 (Laffort, France).

Yeast extract peptone dextrose medium (YPD, 10 g/l yeast extract, 20 g/l peptone, 20 g/l glucose, and 20 g/l agar) was used to inoculate preparation and yeast cell count.

### Laboratory-Scale Fermentation of Wines

Cabernet Sauvignon grapes from a vineyard of the Helan Mountain region in Ningxia of China were destemmed and crushed into must (239.9 g/l total sugar calculated as glucose and 7.1 g/l total acid calculated as tartaric acid, pH 3.96). Eight hundred milliliters of must was filled into a 1.0–l glass bottle, pasteurized at 68.5°C for 30 min. After adding 50 mg/l SO_2_, the must was macerated at 4°C for 12 h, and yeast cells were inoculated at 10^6^ CFU/ml and fermented at 20°C without agitation. Each kind of yeast was inoculated in triplicate.

### Viable Yeast Cell Counting

Samples were taken during fermentation every day and concurrently diluted onto a YPD plate, then incubated at 28°C for 3 days (Suárez et al., 2007). Colonies on the YPD plate were counted as the viable cells of *S. cerevisiae* or non-*Saccharomyces* yeast. Each sample was analyzed in triplicate from the bottles.

### Analytical Determination of Wines

The contents of residual sugar, alcohol, total acid, and volatile acid of wine were analyzed through methods recommended by the International Organization of the Vine and Wine (OIV-MA-AS311-02: R2009, 2009; OIV-MA-AS313-01: R2015, 2015; OIV-MA-AS313-02: R2015, 2015; OIV-MA-AS312-01A: R2016, 2016). The residual sugar contents were expressed as glucose (g/L). The total acid content was expressed as tartaric acid (g/L), and the volatile acid content was expressed as acetic acid (g/L). Each wine sample was measured in triplicates from the bottles.

The extraction of volatile aroma compounds from wine was conducted by headspace solid-phase microextraction with 50/30 μm divinylbenzene carboxen polydimethylsiloxane (DVB/CAR/PDMS) fiber (Supelco, Bellefonte, PA, United States). The extracted volatile compounds were analyzed on an Agilent 6890N gas chromatograph (GC) coupled to an Agilent 5975B mass spectrometer with a DB-5 capillary column (30 m × 0.32 mm × 0.25 μm). An 8-ml sample containing 0.45 g cyclohexanone (internal standard) and 2 g NaCl was put in a 20-ml headspace bottle and stirred by a magnetic bar at 40°C for 15 min. After that, the fiber was exposed to the headspace of bottle for 30 min and immediately desorbed in an injector at 250°C for 3 min. The operating conditions of GC were the following: initial temperature 40°C, increased to 130°C at 3°C/min, then to 250°C at 4°C/min. The injector and detector were set at 250 and 260°C, respectively. The mass spectrometry was operated in electron impact ionization mode at 70 eV, and ion source temperature was 250°C. Detection was carried out in full-scan mode over a range of 30–350 u/s. Compounds were identified by comparing their retention time with MS fragmentation patterns which were obtained from databases Wiley 7.0 and NIST05. All volatile compounds were semi-quantified through the following formula:

$$\begin{array}{c} Compoundcontent\left(mg/mL\right)=\\ \frac{GCpeakareaofcompound\times Quantityofcyclohexanone\left(mg\right)}{GCpeakareaofcyclohexanone\times volumeofsample\left(mL\right)} \end{array}$$

### Sensory Evaluation of Wines

The sensory evaluation was performed as described by Belda et al. (2015) with modification. Twenty milliliters of wine was poured into wine glasses and presented in random order. The preferences for appearance, aroma (fruity, floral, and green), and taste of the wine were scored from 0 (weak) to 9 (intense) by a well-trained panelist (six females and four males) from Huazhong Agricultural University, respectively. The final score of each sensory characteristic was the mean value of 10 scores given by 10 assessors, respectively.

### Data Statistics Processing and Analysis

Data and chart were performed by Microsoft Office 2010 and GraphPad Prism 6.0. One-way ANOVA was completed by SPSS 19.0 software (SPSS Inc., Chicago, IL, United States). Principal component analysis (PCA) was performed by SIMCA-P 14.1 (Umetrics AB, Umea, Sweden).

## Results

### Growth and Sugar Consumption Kinetics of Yeast Strains During Wine Fermentation

**FIGURE 1:**
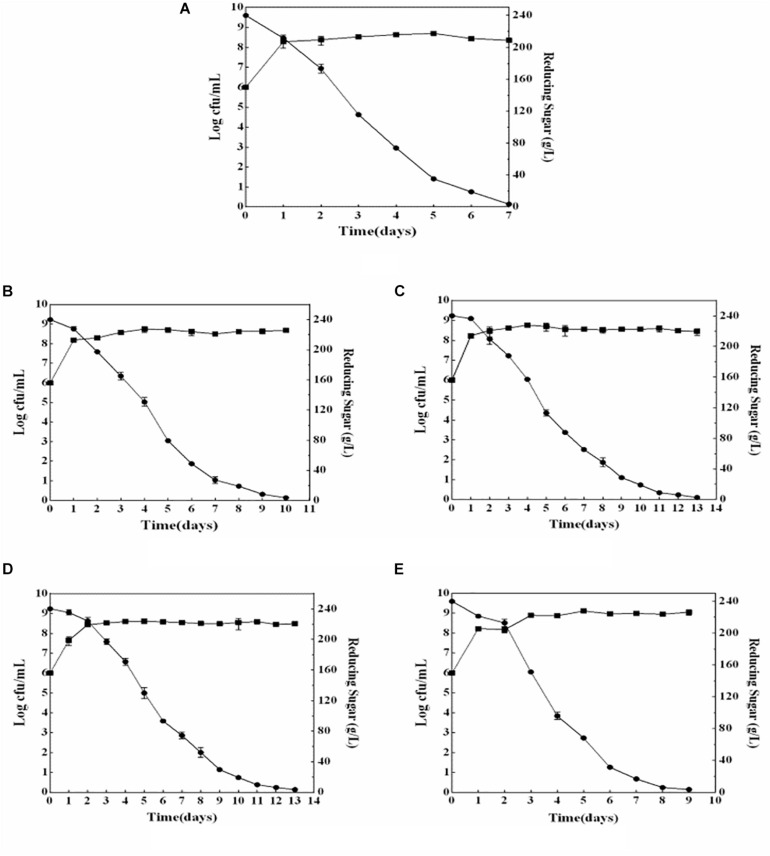
Growth and sugar consumption kinetics of yeast strains during wine fermentation. **(A)** *S. cerevisiae* fermentation; **(B)** *I. terricola* SLY-4 fermentation; **(C)** *P. kudriavzevii* F2-24 fermentation; **(D)** *P. kudriavzevii* F2-16 fermentation; **(E)** *M. pulcherrima* HX-13 fermentation. -■-Growth kinetics of yeast strains -●-Sugar consumption kinetics of yeast strains.

The growth and sugar consumption kinetics of yeast strains indicated that four non-*Saccharomyces* yeast strains could grow normally during wine fermentation (Figure 1). Compared with that of *S. cerevisiae* (2.25 × 10^9^ cells/ml), the biomasses of four non-*Saccharomyces* yeast strains were higher. *M. pulcherrima* HX-13 had the highest biomass (11.45 × 10^9^ cells/ml), followed by *I. terricola* SLY-4 (4.8 × 10^9^ cells/ml), *P. kudriavzevii* F2-16 (3.05 × 10^9^ cells/ml), and *P. kudriavzevii* F2-24 (2.8 × 10^9^ cells/ml). Compared with that of *S. cerevisiae* (7 days), the fermentation periods of the four non-*Saccharomyces* yeasts were longer (9–13 days). *M. pulcherrima* HX-13 had the shortest fermentation period (9 days) among the non-*Saccharomyces* yeasts, followed by *I. terricola* SLY-4 (10 days), *P. kudriavzevii* F2-24 (10 days), and *P. kudriavzevii* F2-16 (13 days).

### Chemical Characteristics of Wines Fermented by Yeast Strains With *β*-Glucosidase Activity

The chemical characteristics of wines fermented by different yeast strains showed that all the fermentations contained 2.71–3.64 g/l residue sugar (expressed as glucose), about 12% ethanol (v/v), 5.13–5.44 g/l total acid (expressed as tartaric acid), and 0.23–0.31 g/l volatile acid (expressed as acetic acid) (Table 1). These results indicated there was no negative effect on the chemical characteristics of wines.

**TABLE 1:** Chemical characteristics of wines fermented by yeast with *β*-glycosidase activity.

Wines	Time (days)	Residual sugar (g/L)	Alcohol content (%, v/v)	Total acid (g/L)	Volatile acid (g/L)
SCw	7	3.57 ± 0.19^a^	12.85 ± 0.36^a^	5.44 ± 0.19^a^	0.23 ± 0.01^b^
SLY-4w	10	3.64 ± 0.07^a^	12.91 ± 0.28^a^	5.44 ± 0.19^a^	0.31 ± 0.01^a^
F2-24w	13	2.71 ± 0.07^b^	12.69 ± 0.08^a^	5.25 ± 0.19^b^	0.26 ± 0.01^b^
F2-16w	13	3.53 ± 0.20^a^	12.08 ± 0.38^a^	5.13 ± 0.18^b^	0.25 ± 0.01^b^
HX-13w	9	3.50 ± 0.30^a^	12.96 ± 0.13^a^	5.31 ± 0.11^b^	0.24 ± 0.00^b^

*Different inline letters in the same row of values indicate significant differences determined by Duncan test at 95% confidence level. SCw, S. cerevisiae fermentation; SLY-4w, I. terricola SLY-4 fermentation; F2-24w, P. kudriavzevii F2-24 fermentation; F2-16w, P. kudriavzevii F2-16 fermentation; HX-13w, M. pulcherrima HX-13 fermentation.*

### Volatile Compounds of Wines Fermented by Yeast Strains With *β*-Glucosidase Activity

The total ion current chromatograms of gas chromatography–mass spectrometry (GC-MS) analysis for all the fermentations indicated that different fermentations had unique chromatogram profiles. Fifty-three kinds of volatile compounds were classified into variety aroma compounds and fermentative aroma compounds. Eleven variety aroma components were clustered into C_6_ compound, terpene, norisoprenoid, and benzene derivative compound. Forty-two fermentative aroma components were clustered into compounds of higher alcohol, fatty acid, ester (acetic ester, fatty acid ethyl ester, and other ester), aldehyde, and ketone (Table 2).

**TABLE 2:** Volatile compounds in different wines (mg/L).

Compounds	Concentration (mg/L)					Odor threshold	OAV	Odors descriptions
	SCw	SLY-4w	F2-24w	F2-16w	HX-13w			
**Varietal aroma**	**85.29 ± 12.88^a^**	**60.16 ± 0.40^b^**	**45.49 ± 6.18^c^**	**62.64 ± 0.53^b^**	**57.89 ± 4.23^b^**			
**C_6_ compounds**	**0.78 ± 0.04^a^**	**0.54 ± 0.02^b^**	**0.21 ± 0.04^d^**	**0.25 ± 0.01^d^**	**0.33 ± 0.00^c^**			
1-Hexanol	0.36 ± 0.05^a^	0.02 ± 0.01^c^	0.03 ± 0.01^c^	0.06 ± 0.02^c^	0.11 ± 0.01^b^	8	<0.1	Green, herb
*cis*-2-Hexen-1-ol	0.42 ± 0.02^c^	0.52 ± 0.03^b^	0.19 ± 0.05^d^	0.20 ± 0.01^d^	0.23 ± 0.01^d^	0.4	>0.1	Green, herb
**Terpenes**	**0.04 ± 0.01^e^**	**0.26 ± 0.01^d^**	**0.59 ± 0.01^b^**	**0.89 ± 0.01^a^**	**0.33 ± 0.04^c^**			
Limonene	0.00 ± 0.00^c^	0.00 ± 0.00^c^	0.15 ± 0.01^b^	0.22 ± 0.01^a^	0.00 ± 0.00^c^	0.1	>1	Lemon, citrus
Linalool	0.01 ± 0.00^e^	0.10 ± 0.01^c^	0.17 ± 0.01^a^	0.12 ± 0.01^b^	0.02 ± 0.01^d^	0.025	>1	Muscat, flowery, fruity
Citronellol	0.02 ± 0.01^d^	0.09 ± 0.01^c^	0.17 ± 0.01^b^	0.42 ± 0.01^a^	0.17 ± 0.01^b^	0.01	>1	Citrus
Nerol	0.01 ± 0.00^d^	0.08 ± 0.02^c^	0.11 ± 0.01^b^	0.14 ± 0.02^a^	0.14 ± 0.03^a^	0.03	>1	Citrus
**C_13_-Norisoprenoids**	**0.00 ± 0.00^c^**	**0.03 ± 0.01^a^**	**0.01 ± 0.00^b^**	**0.02 ± 0.01^b^**	**0.00 ± 0.00^c^**			
*β*-Ionone	0.00 ± 0.00^c^	0.03 ± 0.01^a^	0.01 ± 0.00^b^	0.02 ± 0.01^b^	0.00 ± 0.00^c^	9*10^−5^	>1	Raspberry, violet, sweet fruity
**Benzene derivatives**	**84.51 ± 12.91^a^**	**59.34 ± 0.41^b^**	**44.68 ± 6.21^c^**	**61.49 ± 0.56^b^**	**57.23 ± 4.27^b^**			
Benzaldehyde	0.29 ± 0.06^b^	0.81 ± 0.02^a^	0.00 ± 0.00^c^	0.00 ± 0.00^c^	0.87 ± 0.06^a^	2	<0.1	
Benzyl alcohol	0.00 ± 0.00^b^	0.30 ± 0.01^a^	0.00 ± 0.00^b^	0.00 ± 0.00^b^	0.00 ± 0.00^b^	200	<0.1	Almond, fatty
Phenethyl alcohol	72.16 ± 11.14^a^	30.99 ± 1.56^d^	40.35 ± 6.09^c^	55.14 ± 0.75^b^	33.24 ± 4.92^d^	14	>1	Rose, soft tommy
Phenylethyl acetate	12.07 ± 1.71^c^	27.25 ± 2.00^a^	4.33 ± 0.12^d^	6.35 ± 0.20^d^	23.13 ± 0.59^b^	0.25	>1	Floral, rose
**Fermentative aroma**	**404.89 ± 1.45^d^**	**499.00 ± 9.53^c^**	**530.83 ± 14.26^b^**	**524.77 ± 23.19^b^**	**636.72 ± 12.92^a^**			
**Higher alcohols**	**202.03 ± 1.93^e^**	**233.21 ± 1.86^d^**	**273.88 ± 7.06^c^**	**285.47 ± 4.60^b^**	**308.67 ± 10.16^a^**			
1-Propanol	2.51 ± 0.17^c^	6.89 ± 0.02^b^	2.85 ± 0.49^c^	8.30 ± 0.41^a^	0.00 ± 0.00^d^	306	<0.1	Fresh, alcohol
Isobutyl alcohol	8.06 ± 0.61^d^	10.73 ± 0.52^d^	29.50 ± 1.06^c^	61.01 ± 4.47^b^	73.57 ± 3.23^a^	40		Mild sweet, alcohol
1-Butanol	0.41 ± 0.00^c^	0.85 ± 0.17^ab^	0.69 ± 0.13^b^	1.00 ± 0.15^a^	0.00 ± 0.00^d^	150	<0.1	Medicinal, fusel, pungency
Isoamyl alcohol	120.46 ± 3.21^b^	167.28 ± 0.05^a^	172.69 ± 7.57^a^	109.57 ± 3.90^c^	169.06 ± 1.60^a^	30	>1	Alcohol, harsh, bitter, banana
2-Methyl-1-butanol	68.74 ± 0.50^b^	45.36 ± 1.20^c^	66.97 ± 0.00^b^	102.68 ± 3.11^a^	64.47 ± 14.99^b^	65	>0.1	
2,3-Butanediol	0.77 ± 0.04^c^	1.26 ± 0.01^b^	0.64 ± 0.19^c^	1.67 ± 0.21^a^	1.44 ± 0.02^b^	120	<0.1	Butter, creamy, chemical
3-Methyl-1-pentanol	0.56 ± 0.10^a^	0.36 ± 0.01^b^	0.00 ± 0.00^c^	0.00 ± 0.00^c^	0.00 ± 0.00^c^	0.5	>1	Fusel
1-Octanol	0.27 ± 0.03^c^	0.50 ± 0.05^b^	0.44 ± 0.01^b^	1.26 ± 0.15^a^	0.14 ± 0.03^d^	0.9	0.1-1	Waxy
1-Decanol	0.27 ± 0.08^a^	0.00 ± 0.00^c^	0.12 ± 0.01^d^	0.00 ± 0.00^c^	0.00 ± 0.00^c^	0.4	0.1-1	Citrus, fatty
**Fatty acids**	**5.11 ± 0.06^c^**	**9.16 ± 0.33^a^**	**4.44 ± 0.14^d^**	**5.91 ± 0.32^b^**	**5.28 ± 0.22^c^**			
Isobutyric acid	0.18 ± 0.01^b^	0.00 ± 0.00^c^	0.00 ± 0.00^c^	0.68 ± 0.03^a^	0.00 ± 0.00^c^	8.1	<0.1	Phenol, chemical, fatty
2-Methylbutyric acid	0.32 ± 0.04^b^	0.00 ± 0.00^c^	0.29 ± 0.00^b^	1.26 ± 0.11^a^	0.00 ± 0.00^c^	0.25	>1	Cheese
Isopentanoic acid	0.00 ± 0.00^c^	0.63 ± 0.07^b^	0.47 ± 0.02^b^	1.02 ± 0.49^a^	0.00 ± 0.00^c^	0.033	>1	Sweaty feet
Hexanoic acid	1.54 ± 0.06^b^	2.21 ± 0.03^a^	1.52 ± 0.06^b^	1.13 ± 0.00^d^	2.25 ± 0.10^a^	0.42	>1	Cheese, rancid
Octanoic acid	3.01 ± 0.04^c^	5.84 ± 0.20^a^	2.06 ± 0.05^e^	1.83 ± 0.04^f^	2.57 ± 0.11^d^	0.5	>1	Rancid, harsh, cheese, fatty acid
Decanoic acid	0.06 ± 0.00^cd^	0.48 ± 0.03^b^	0.11 ± 0.01^c^	0.00 ± 0.00^d^	0.46 ± 0.01^b^	1	<0.1	Unpleasant
**Esters**	**194.88 ± 3.24^e^**	**251.99 ± 10.90^b^**	**250.04 ± 21.92^b^**	**231.50 ± 19.11^b^**	**319.98 ± 23.37^a^**			
**Acetic esters**	**90.82 ± 3.40^c^**	**203.12 ± 4.25^b^**	**217.94 ± 25.24^ab^**	**195.87 ± 14.59^b^**	**242.58 ± 17.28^a^**			
Ethyl acetate	50.46 ± 2.14^d^	107.46 ± 8.00^bc^	143.02 ± 10.44^a^	112.46 ± 11.00^b^	94.47 ± 11.00^c^	7.5	>1	Fruity, sweet
Propyl acetate	0.00 ± 0.00^f^	0.41 ± 0.00^c^	0.33 ± 0.01^d^	1.13 ± 0.00^a^	0.70 ± 0.02^b^	4.7	<1	Fruity
Isobutyl acetate	0.63 ± 0.07^d^	1.27 ± 0.15^c^	0.82 ± 0.05^d^	2.53 ± 0.40^b^	10.23 ± 0.26^a^	1.6	>0.1	Garnetberry, fruity, flowery
Isoamyl acetate	33.70 ± 0.94^d^	85.91 ± 5.35^bc^	71.47 ± 14.98^b^	75.31 ± 3.15^c^	117.79 ± 6.00^a^	0.03	>1	Banana
2-Methylbutyl acetate	5.78 ± 2.11^bc^	7.53 ± 1.32^b^	2.30 ± 0.12^d^	4.45 ± 0.05^c^	19.39 ± 0.00^a^	0.02–0.05	>1	Fruity
Pentanol acetate	0.00 ± 0.00^b^	0.18 ± 0.05^a^	0.00 ± 0.00^b^	0.00 ± 0.00^b^	0.00 ± 0.00^b^			
3-Methylpentyl acetate	0.00 ± 0.00^b^	0.37 ± 0.07^a^	0.00 ± 0.00^b^	0.00 ± 0.00^b^	0.00 ± 0.00^b^			
Hexyl acetate	0.19 ± 0.03^b^	0.00 ± 0.00^c^	0.00 ± 0.00^c^	0.00 ± 0.00^c^	0.00 ± 0.00^c^	1.5	0.1–1	Pleasant fruity, pear
Octyl acetate	0.06 ± 0.00^b^	0.00 ± 0.00^c^	0.00 ± 0.00^c^	0.00 ± 0.00^c^	0.00 ± 0.00^c^			
**Fatty acid ethyl esters**	**101.42 ± 0.20^a^**	**48.87 ± 6.65^c^**	**32.11 ± 3.32^d^**	**35.63 ± 4.52^d^**	**77.40 ± 6.09^b^**			
Ethyl propanoate	0.25 ± 0.02^d^	0.00 ± 0.00^e^	0.48 ± 0.02^c^	1.48 ± 0.25^a^	1.10 ± 0.06^b^	1.8–2.1	0.1–1	
Ethyl butyrate	2.35 ± 0.10^b^	1.81 ± 0.54^bc^	1.45 ± 0.39^c^	2.10 ± 0.09^b^	5.47 ± 0.50^a^	0.02	>1	Sour fruit, strawberry, fruity
Ethyl hexanoate	44.99 ± 0.34^a^	12.43 ± 2.10^d^	11.24 ± 1.09^d^	10.67 ± 1.22^d^	31.77 ± 2.32^b^	0.014	>1	Green apple, fruity, strawberry, anise
Ethyl heptanoate	38.78 ± 0.54^a^	16.47 ± 1.12^c^	11.58 ± 1.02^d^	11.95 ± 0.70^d^	23.47 ± 2.42^b^	0.005	>1	Fruity, sweet, anise, wax
Ethyl octanoate	0.06 ± 0.01^b^	0.00 ± 0.00^c^	0.00 ± 0.00^c^	0.00 ± 0.00^c^	0.00 ± 0.00^c^	1.3	<0.1	banana
Ethyl non-anoate	1.19 ± 0.06^a^	0.19 ± 0.03^c^	0.00 ± 0.00^c^	0.00 ± 0.00^c^	0.43 ± 0.02^b^	0.1	>1	Green, fruity, fatty
Ethyl 9-decenoate	8.09 ± 0.26^c^	12.39 ± 1.71^b^	4.67 ± 0.55^d^	5.06 ± 1.91^d^	9.65 ± 0.49^c^	0.1	>1	Fruity, fatty, pleasant, wax flavor
Ethyl decanoate	4.04 ± 0.62^b^	3.48 ± 0.83^b^	1.73 ± 0.14^c^	2.16 ± 0.15^c^	3.26 ± 0.29^b^	1.5	>1	Waxy
Ethyl dodecanoate	0.64 ± 0.05^b^	0.59 ± 0.03^c^	0.18 ± 0.01^e^	0.75 ± 0.01^a^	0.30 ± 0.02^d^	2	0.1–1	Fatty, butter
Ethyl myristate	0.76 ± 0.03^b^	0.63 ± 0.18^b^	0.36 ± 0.09^c^	0.34 ± 0.08^c^	1.01 ± 0.01^a^	100–200	<0.1	Green, fruity
Diethyl succinate	0.30 ± 0.01^d^	0.90 ± 0.12^b^	0.44 ± 0.03^c^	1.14 ± 0.13^a^	0.98 ± 0.01^b^	1.5	0.1–1	Fatty
Ethyl palmitate	38.78 ± 0.54^a^	16.47 ± 1.12^c^	11.58 ± 1.02^d^	11.95 ± 0.70^d^	23.47 ± 2.42^b^	0.005	>1	Fruity, sweet, anise, wax
**Other esters**	**2.64 ± 0.04^a^**	**0.00 ± 0.00^c^**	**0.00 ± 0.00^c^**	**0.00 ± 0.00^c^**	**0.00 ± 0.00^c^**			
Isoamyl octanoate	1.29 ± 0.06^a^	0.00 ± 0.00^c^	0.00 ± 0.00^c^	0.00 ± 0.00^c^	0.00 ± 0.00^c^	0.13	>1	Sweet, cheese
Isoamyl hexanoate	1.35 ± 0.10^a^	0.00 ± 0.00^c^	0.00 ± 0.00^c^	0.00 ± 0.00^c^	0.00 ± 0.00^c^			
**Carbonyl compounds**	**2.89 ± 0.06^b^**	**4.64 ± 0.16^a^**	**2.48 ± 0.46^b^**	**1.90 ± 0.21^c^**	**2.79 ± 0.51^b^**			
1-Non-anal	0.17 ± 0.01^b^	0.00 ± 0.00^d^	0.27 ± 0.03^a^	0.00 ± 0.00^d^	0.00 ± 0.00^d^	0.015	>1	Waxy
1-Decanal	0.32 ± 0.03^b^	0.23 ± 0.05^c^	0.00 ± 0.00^e^	0.00 ± 0.00^e^	0.62 ± 0.01^a^	0.001	>1	Sweet
2,3-Butanedione	0.94 ± 0.02^d^	3.16 ± 0.05^a^	1.52 ± 0.07^b^	1.09 ± 0.06^c^	1.04 ± 0.05^c^	0.1	>1	Butter, cheese
2,3-Pentanedione	1.46 ± 0.01^a^	1.26 ± 0.07^ab^	0.70 ± 0.36^c^	0.81 ± 0.27^bc^	1.14 ± 0.47^abc^	<0.1	>1	Butter, cheese

*Data show average of triplicates ± SD. Different letters indicated differences among wines determined by Duncan test at the 95% confidence level. SCw, S. cerevisiae fermentation; SLY-4w, I. terricola SLY-4 fermentation; F2-24w, P. kudriavzevii F2-24 fermentation; F2-16w, P. kudriavzevii F2-16 fermentation; HX-13w, M. pulcherrima HX-13 fermentation. Bold values represent the total content of a class of substances.*

The effects of non-*Saccharomyces* yeast strains on the aroma compounds were evaluated as follows.

**FIGURE 2:**
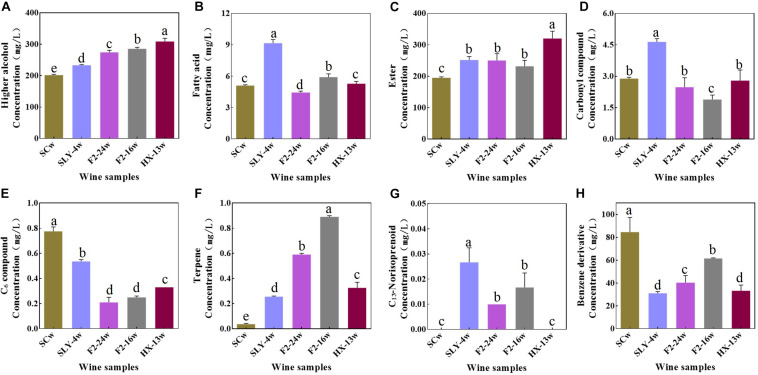
Concentration of aroma compounds in wines fermented by different yeast strains. **(A)** Higher alcohols. **(B)** Fatty acids. **(C)** Esters. **(D)** Carbonyl compounds. **(E)** C_6_ compounds. **(F)** Terpenes. **(G)** C_13_-Norisoprenoids. **(H)** Benzene derivatives; SCw, *S. cerevisiae* fermentation; SLY-4w, *I. terricola* SLY-4 fermentation; F2-24w, *P. kudriavzevii* F2-24 fermentation; F2-16w, *P. kudriavzevii* F2-16 fermentation; HX-13w, *M. pulcherrima* HX-13 fermentation.

Compared with those of the *S. cerevisiae* single fermentation, the total contents of varietal aroma compounds in non-*Saccharomyces* yeast fermentations were lower (45.49–62.64 mg/l). Among the varietal aroma compounds, lower contents of C_6_ compounds (0.21–0.54 mg/l) and benzene derivative compounds (44.68–61.49 mg/l) and higher contents of terpene (0.26–0.89 mg/l) and C_13_-norisoprenoid compounds (0.01–0.03 mg/l) were produced. Limonene, linalool, citronellol, nerol, *β*-ionone, phenylethyl alcohol, and phenylethyl acetate were the main odor active variety aroma compounds (OAV > 1) (Table 2 and Figure 2).

Compared with the *S. cerevisiae* single fermentation, the four non-*Saccharomyces* yeast fermentations contained higher contents of fermentative aroma compounds (499.00–636.72 mg/l) (Table 2 and Figure 2), especially higher alcohol (233.21–308.67 mg/l), and ester compounds (250.04–319.98 mg/l), and they produced higher concentrations of acetate compounds (195.87–242.58 mg/l) and lower concentrations of fatty acid ethyl ester compounds (32.11–77.40 mg/l). Isoamyl alcohol, isobutanol, 2-methylpentanol, 1-octanal, ethyl acetate, isobutyl acetate, isoamyl acetate, 2-methylbutyl acetate, ethyl butyrate, ethyl hexanoate, ethyl octanoate, ethyl 9-decenoate, ethyl decanoate, ethyl laurate, isoamyl caprylate, and isoamyl caproate were the main odor active fermentation aroma compounds.

### PCA of Volatile Compounds From Wines Fermented by Yeast Strains With *β*-Glucosidase Activity

**FIGURE 3:**
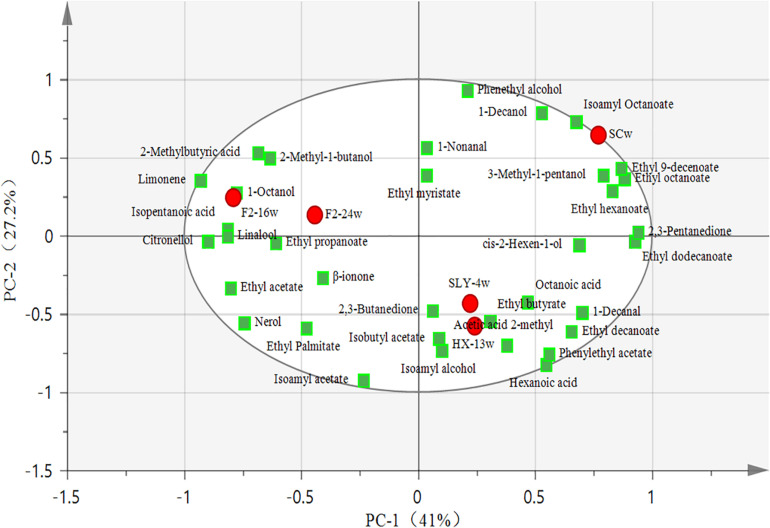
PCA plot of volatile compounds from wines fermented by different yeast strains. ● Wine samples; ■ Aroma compounds; SCw, *S. cerevisiae* fermentation; SLY-4w, *I. terricola* SLY-4 fermentation; F2-24w, *P. kudriavzevii* F2-24 fermentation; F2-16w, *P. kudriavzevii* F2-16 fermentation; HX-13w, *M. pulcherrima* HX-13 fermentation.

A principal component analysis (PCA) was carried out to reveal the correlation and segregation of volatile compounds with different yeast strain fermentations. Here 68.2% of variance was explained, and PC1 and PC2 accounted for 41% and 27.2% of variance, respectively. The *P. kudriavzevii* F2-16 fermentation and the *P. kudriavzevii* F2-24 fermentation were mainly grouped with varietal aroma compounds such as limonene, linalool, citronellol, and some kinds of fermentative aroma compounds such as 1-octanol, 2-methyl-1-butanol, isopentanoic acid, and 2-methylbutyric acid. The *I. terricola* SLY-4 fermentation and the *M. pulcherrima* HX-13 fermentation were closely clustered with various fermentative aroma compounds such as 2,3-butanediol, isoamyl alcohol, isobutyl acetate, acetic acid 2-methyl, ethyl butyrate, octanoic acid, 1-decanol, ethyl decanoate, phenylethyl acetate, and hexanoic acid. *S. cerevisiae* fermentation was grouped with some fermentative aroma compounds such as isoamyl octanoate, 3-methyl-1-pentanol, ethyl 9-decenoate, ethyl octanoate, and ethyl hexanoate (Figure 3).

### Sensory Evaluation of Wines Fermented by Different Yeast Strains With *β*-Glucosidase Activity

**FIGURE 4:**
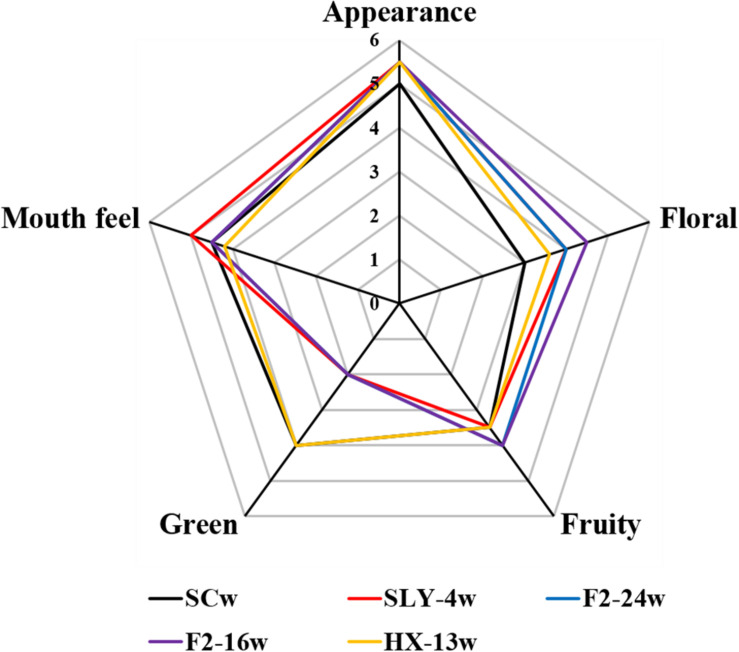
Aroma compounds profiles of wines fermented by different yeast strains with *β*-glucosidase activity. SCw, *S. cerevisiae* fermentation; SLY-4w, *I. terricola* SLY-4 fermentation; F2-24w, *P. kudriavzevii* F2-24 fermentation; F2-16w, *P. kudriavzevii* F2-16 fermentation; HX-13w, *M. pulcherrima* HX-13 fermentation.

Compared with that of the *S. cerevisiae* single fermentation, all the non-*Saccharomyces* yeast fermentations had no significant difference in appearance, but they had a stronger fruity and floral flavor and a weaker green flavor with exception of *M. pulcherrima* HX-13 fermentation. *I. terricola* SLY-4 fermentation had the best taste, followed by the fermentations of *S. cerevisiae*, *P. kudriavzevii* F2-24, *P. kudriavzevii* F2-16, and *M. pulcherrima* HX-13. The order of total acceptance for the wines from high to low was fermentation of *I. terricola* SLY-4, *P. kudriavzevii* F2-24, *P. kudriavzevii* F2-16, *M. pulcherrima* HX-13, and *S. cerevisiae* (Figure 4).

## Discussion

Compared with those of the *S. cerevisiae* single fermentation, the four non-*Saccharomyces* yeast strains had higher biomasses and longer fermentation periods which were also reported by Andorrà et al. (2010) and Belda et al. (2015). Those results mean that the four non-*Saccharomyces* yeast strains consumed sugar more slowly than *S. cerevisiae* did, but they could consume it completely.

Compared with *S. cerevisiae*, the four non-*Saccharomyces* yeast strains had no negative effects on the chemical characteristics of wines. Specifically, they produced lower contents of C_6_ compounds and benzene derivative and higher contents of terpene, *β*-ionone, higher alcohol, and ester compounds, these phenomena were also reported by Nyanga et al. (2013); Del Mónaco et al. (2014), Lu et al. (2017), and Sun et al. (2017). Low concentrations of C_6_ compounds would reduce the green flavor of wines (Mendez-Costabel et al., 2014; Vilanova et al., 2016), while high contents of terpene, isoprenoid, benzene derivative, ester, and higher alcohol would enhance the fruity and floral flavor of wines (Pretorius and Lambrechts, 2000; Swiegers and Pretorius, 2005; Noguerol-Pato et al., 2012; Sun et al., 2017). The sensory evaluation results of wines indeed indicated that the green flavor of wines was reduced by the four non-*Saccharomyces* yeast strains with exception of *M. pulcherrima* HX-13, and their fruity and floral flavors were enhanced. More importantly, the first report about *M. pulcherrima* could produce high content of varietal aroma compounds in wine. The volatile compound profiles of the four non-*Saccharomyces* yeast fermentations were significantly different from those of *S. cerevisiae* fermentation. Moreover, the volatile compound profiles of *P. kudriavzevii* F2-16 and *P. kudriavzevii* F2-24 fermentations were remarkably different from those of *I. terricola* SLY-4 and *M. pulcherrima* HX-13 fermentations. Different volatile compounds profiles would take different flavor characteristics on wines (Lu et al., 2017; Siebert et al., 2018), which was consistent with sensory evaluation results of wines.

## Conclusion

Compared with those of *S. cerevisiae* single fermentation, the four non-*Saccharomyces* yeast strains could grow and consume sugar completely and had no significantly negative effect on chemical characteristics of wines. All the four non-*Saccharomyces* yeast strains could improve the flavor and quality of wines. Moreover, different yeast strains produced different aroma compounds profiles and take on different aroma characteristics of wines. The four non-*Saccharomyces* yeast strain fermentations received higher acceptance of sensory evaluation than *S. cerevisiae* did, and *I. terricola* SLY-4 fermentation got the highest sensory evaluation score, followed by *P. kudriavzevii* F2-24, *P. kudriavzevii* F2-16, and *M. pulcherrima* HX-13 fermentation from high to low. However, pure non-*Saccharomyces* yeast fermentation had disadvantages with long fermentation periods and lower content of benzene derivative and fatty acid ethyl ester compounds. To overcome the disadvantages of pure non-*Saccharomyces* yeast fermentation, co-fermentation of several non-*Saccharomyces* yeast strains with different aroma compound profiles, or pure *S. cerevisiae* fermentation of must with addition of complex *β*-glucosidase (crude or purified) from different non-*Saccharomyces* yeast strains, might be used to further improve the kind of aroma compounds and the flavor complexity of wine and shorten the fermentation period of wine. The research results will provide non-Saccharomyces yeast strains to improve the flavor and quality of wines, and a reference for the selection of other non-*Saccharomyces* yeasts strains with better oenological characteristics.

## Data Availability Statement

The original contributions presented in the study are included in the article/supplementary material, further inquiries can be directed to the corresponding author.

## Author Contributions

XZ and YZ contributed to the experimental design. YZ, WZ, and TQ completed the experiments, performed statistical analysis, and wrote the manuscript. TQ and JL contributed to the experiment verification. JL, YZ, and TQ contributed to the revision of the manuscript. All authors contributed to the article and approved the submitted version.

## Conflict of Interest

The authors declare that the research was conducted in the absence of any commercial or financial relationships that could be construed as a potential conflict of interest.

## Publisher’s Note

All claims expressed in this article are solely those of the authors and do not necessarily represent those of their affiliated organizations, or those of the publisher, the editors and the reviewers. Any product that may be evaluated in this article, or claim that may be made by its manufacturer, is not guaranteed or endorsed by the publisher.
